# Intra-articular injection of two different doses of autologous bone marrow mesenchymal stem cells versus hyaluronic acid in the treatment of knee osteoarthritis: long-term follow up of a multicenter randomized controlled clinical trial (phase I/II)

**DOI:** 10.1186/s12967-018-1591-7

**Published:** 2018-07-31

**Authors:** José María Lamo-Espinosa, Gonzalo Mora, Juan F. Blanco, Froilán Granero-Moltó, Jorge María Núñez-Córdoba, Silvia López-Elío, Enrique Andreu, Fermín Sánchez-Guijo, José Dámaso Aquerreta, José María Bondía, Andrés Valentí-Azcárate, María del Consuelo del Cañizo, Eva María Villarón, Juan Ramón Valentí-Nin, Felipe Prósper

**Affiliations:** 1 Department of Orthopaedic Surgery and Traumatology, Clínica Universidad de Navarra, Pamplona, Spain; 2 Cell Therapy Area, Clínica Universidad de Navarra, Pamplona, Spain; 3 Department of Traumatology, Complejo Hospitalario de Salamanca, Salamanca, Spain; 4 Department of Hematology, Complejo Hospitalario de Salamanca, Salamanca, Spain; 5 Department of Radiology, Clínica Universidad de Navarra, Pamplona, Spain; 6 Division of Biostatistics, Research Support Service, Central Clinical Trials Unit, University of Navarra Clinic, Pamplona, Spain; 7 Department of Preventive Medicine and Public Health, Medical School, University of Navarra, Pamplona, Spain; 8 Epidemiology and Public Health Area, Navarra Institute for Health Research (IdiSNA), Pamplona, Spain; 9 Department of Hematology, Clínica Universidad de Navarra, Pamplona, Spain

**Keywords:** Knee osteoarthritis, Mesenchymal stem cells, Intraarticular injection

## Abstract

**Background:**

Mesenchymal stromal cells (MSCs) are a promising option to treat knee osteoarthritis (OA). Their safety and usefulness have been reported in several short-term clinical trials but less information is available on the long-term effects of MSC in patients with osteoarthritis. We have evaluated patients included in our previous randomized clinical trial (CMM-ART, NCT02123368) to determine their long-term clinical effect.

**Materials:**

A phase I/II multicenter randomized clinical trial with active control was conducted between 2012 and 2014. Thirty patients diagnosed with knee OA were randomly assigned to Control group, intraarticularly administered hyaluronic acid alone, or to two treatment groups, hyaluronic acid together with 10 × 10^6^ or 100 × 10^6^ cultured autologous bone marrow-derived MSCs (BM-MSCs), and followed up for 12 months. After a follow up of 4 years adverse effects and clinical evolution, assessed using VAS and WOMAC scorings are reported.

**Results:**

No adverse effects were reported after BM-MSCs administration or during the follow-up. BM-MSCs-administered patients improved according to VAS, median value (IQR) for Control, Low-dose and High-dose groups changed from 5 (3, 7), 7 (5, 8) and 6 (4, 8) to 7 (6, 7), 2 (2, 5) and 3 (3, 4), respectively at the end of follow up (Low-dose vs Control group, p = 0.01; High-dose vs Control group, p = 0.004). Patients receiving BM-MSCs also improved clinically according to WOMAC. Control group showed an increase median value of 4 points (− 11;10) while Low-dose and High-dose groups exhibited values of − 18 (− 28;− 9) and − 10 (− 21;− 3) points, respectively (Low-dose vs Control group p = 0.043). No clinical differences between the BM-MSCs receiving groups were found.

**Conclusions:**

Single intraarticular injection of in vitro expanded autologous BM-MSCs is a safe and feasible procedure that results in long-term clinical and functional improvement of knee OA.

## Background

Osteoarthritis is a chronic disease involving the progressive degeneration of the articular cartilage and subchondral bone, accompanied by synovitis [1]. Current treatment options for articular cartilage injury and osteoarthritis are aimed to relieve inflammation and pain, but have no effect on the natural progression of the disease [2]. Mesenchymal stromal cells are a promising option to treat knee osteoarthritis (OA) where, to date, knee arthroplasty is the only therapeutic option [3]. In the short term, the safety and usefulness of single injection of expanded autologous MSCs have been reported with positive results [4–6]. However, long-term results on the efficacy of MSCs in patients with osteoarthritis have been scarcely reported. Reservations about the time and extent of the anti-inflammatory effects of MSCs are present, questioning the real value of these therapies in the medium and long term.

Here, we present the long-term results of a prospective randomized clinical trial (No EudraCT: 2009-017624-72, Clinical Trials. gov identifier: NCT02123368) of patients with knee osteoarthritis previously reported [4]. The occurrence of complications and/or adverse effects during the clinical study was registered. The knee OA treatments received during these time were recorded. In addition, the response to the intra-articular infusion of HA with or without BM-MSCs was assessed using VAS and WOMAC scores in the patients whom did not underwent total knee arthroplasty.

Because the mild effect reported in MRI studies during the initial follow-up, 12 months, and because the absence of femorotibial joint space in 50% of the patients, 0 mm at baseline (IV Kellgren-Lawrence grade), imaging studies were not prolonged.

## Demographic data

**Fig. 1:**
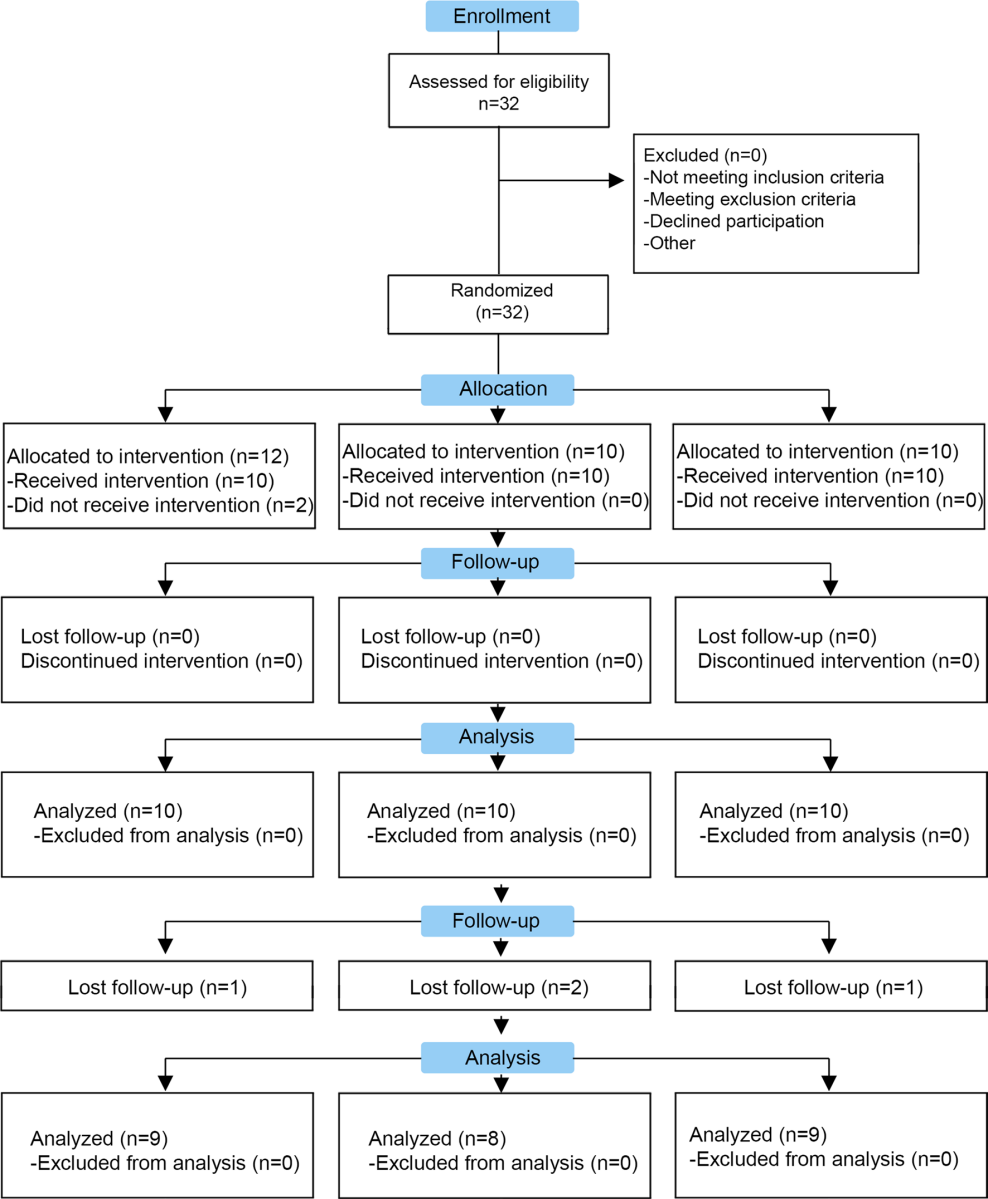
Study flow diagram. We have included 27 patients of the 30 patients that participate in the clinical trial

We were able to contact 27 of the 30 patients included in the clinical trial and them have been included in the follow up (Fig. 1). Two of the patients of the Control group and one patient of the Low-dose group received a total knee arthroplasty. Nonetheless, one of these patients of the Control group was included in the clinical analysis because the surgical treatment was performed after the data collection. In addition, two patients of the Control group underwent infiltration treatment with platelet rich plasma in the knee included in the clinical trial. In spite of these, we have finally included 25 patients (9, 8 and 8 patients in Control, Low-dose and High-dose group, respectively) for the clinical analysis (Fig. 1). All the groups showed similar baseline characteristics of age and body mass index. Patients in the three groups showed an uneven distribution according to the Kellgren-Lawrence scale but without statistical significance (p = 0.585, Table 1). The follow up was 48 months (4 years).

**Table 1:** Demographic data

	Control	BM-MSCs	
		Low-dose	High-dose
N	9	8	8
Age (years)	60.6 (58.9, 61.1)	65.9 (58.3, 69.5)	57.8 (54.4, 63.0)
Males, n (%)	7 (77.8)	4 (50)	6 (75)
BMI (kg/m^2^)	29.4 (26.2, 30.8)	26.6 (23.6, 32)	28.6 (24.9, 31.8)
K-L* 2, n (%)	4 (44.4)	1 (12.5)	2 (25)
K-L* 3, n (%)	2 (22.2)	2 (25)	3 (37.5)
K-L* 4, n (%)	3 (33.3)	5 (62.5)	4 (37.5)

Data are presented as median [interquartile range (IQR)]. OA osteoarthritis *K-L: Kellgren and Lawrence grading scale of severity of knee osteoarthritis at the beginning of the clinical trial

### Safety

No serious adverse events or complications derived from the procedures or treatments were noted during the follow up. The patients who required anti-inflammatory treatment during the first 24 h after infiltration did not evolve with greater pain at the end of follow up.

### Clinical assessment of pain and function

VAS and WOMAC clinical scores were used in order to obtain the best picture of how patients perceived their own evolution at 4 years.

**Fig. 2:**
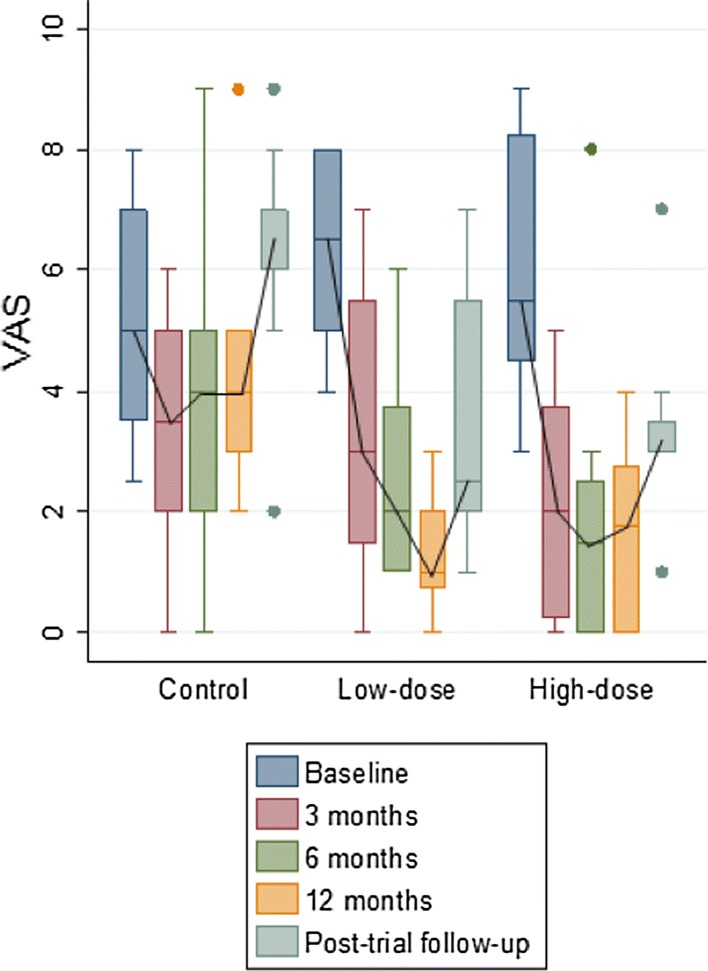
VAS scores along the study. The median values of VAS in the three groups before administration of treatments and 3, 6, 12 months and 4 years afterwards are presented. At 4 years: Low-dose vs Control group, p = 0.01 and High-dose vs Control group, p = 0.004

The VAS scale showed a progressive improvement during the follow up in the groups treated with BM-MSCs (Fig. 2) while the control group, patients showed a progressive deterioration, increasing in two points the median at the end of the follow up. Median VAS values (IQR) for Control, Low-dose and High-dose groups changed from 5 (3, 7), 7 (5, 8) and 6 (4, 8) to 7 (6, 7), 2 (2, 5) and 3 (3, 4), respectively at the end of follow up (Low-dose vs Control group, p = 0.01; High-dose vs Control group, p = 0.004).

**Fig. 3:**
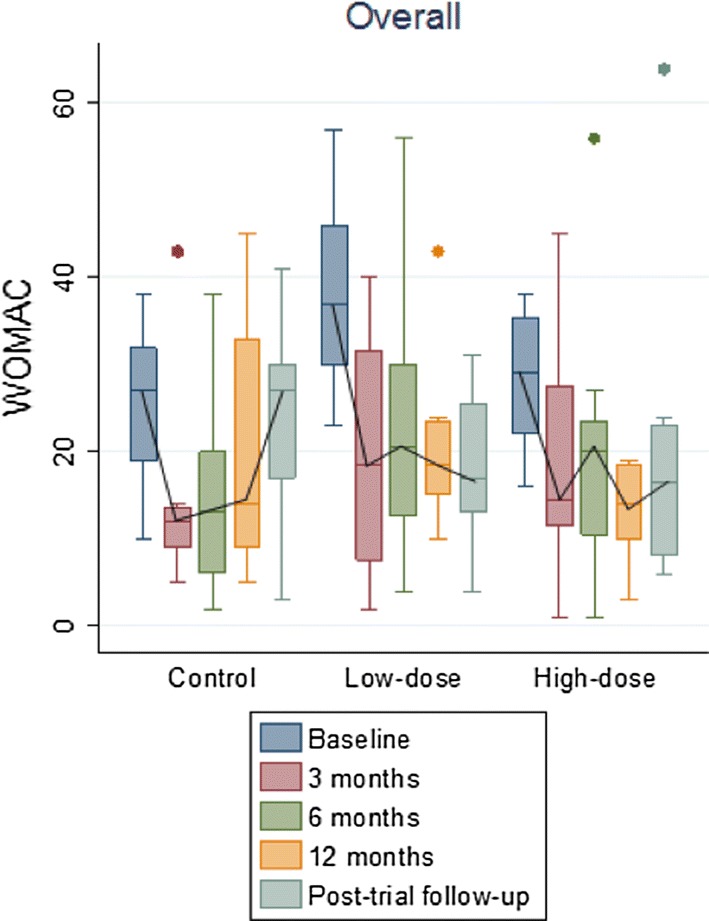
WOMAC scores along the study. The median values of WOMAC in the three groups before administration of treatments and 3, 6, 12 months and 4 years afterwards are presented. At 4 years: Low-dose vs Control group, p = 0.01 and High-dose vs Control group, ns

Similarly, the results of the WOMAC score showed an improvement at the end of the follow up in both groups treated with BM-MSCs. Median WOMAC values (IQR) for Control, Low-dose and High-dose groups changed from 27 (19, 32), 37 (30, 46) and 29 (22, 35.5) to 27 (17, 30), 17 (13, 25.5) and 16.5 (8, 23), respectively at the end of follow up (Low-dose vs Control group, p = 0.04). Furthermore, although patients receiving only HA initially perceived some improvement for pain and physical function subscores, this perception was not sustained after long-term follow up. Intraarticular delivery of BM-MSCs, especially when used at low dose, enabled patients to perceive an improvement in their perception of pain in their daily activity (Fig. 3).

A statistically significant improvement in WOMAC value (calculated as the value at baseline versus end of follow up) was observed in patients receiving BM-MSCs, but not in the group treated with HA alone [4 (− 11, 10), − 18 (− 27.5, 8.5), and − 10 (− 21.5, − 3), median (IQR), for Control, Low-dose and High-dose BM-MSCs groups, respectively]. Thus, only the patients who had been treated with BM-MSCs met criteria to be considered WOMAC responders after 4 years of follow up [7].

## Conclusions

Our study shows that the single intraarticular injection of in vitro expanded autologous BM-MSCs together with HA is a safe and feasible procedure that results in a clinical and functional improvement of knee OA after a follow up of 4 years. There are some questions that would need further analysis, especially if a high dose of cells is needed and if the repeated intraaticular injection of BM-MSC may increase clinical results. In any case these results support the development of future phase III clinical trial.

## Abbreviations

BM-MSCs: bone marrow mesenchymal stromal cells
GMP: good manufacture practices
HA: hyaluronic acid
K-L: Kellgren and Lawrence scale
MRI: magnetic resonance imaging
MSCs: mesenchymal stromal cells
OA: osteoarthritis
VAS: visual analogue scale
WOMAC: Western Ontario and McMaster Universities Osteoarthritis Index
